# Kraken: ultrafast metagenomic sequence classification using exact alignments

**DOI:** 10.1186/gb-2014-15-3-r46

**Published:** 2014-03-03

**Authors:** Derrick E Wood, Steven L Salzberg

**Affiliations:** 1 Department of Computer Science and Center for Bioinformatics and Computational Biology, University of Maryland, College Park, MD, USA; 2 Center for Computational Biology, McKusick-Nathans Institute of Genetic Medicine, Johns Hopkins University School of Medicine, Baltimore, MD, USA; 3 Department of Biostatistics, Bloomberg School of Public Health, Johns Hopkins University, Baltimore, MD, USA

**Keywords:** metagenomics, sequence classification, sequence alignment, next-generation sequencing, microbiome

## Abstract

Kraken is an ultrafast and highly accurate program for assigning taxonomic labels to metagenomic DNA sequences. Previous programs designed for this task have been relatively slow and computationally expensive, forcing researchers to use faster abundance estimation programs, which only classify small subsets of metagenomic data. Using exact alignment of *k*-mers, Kraken achieves classification accuracy comparable to the fastest BLAST program. In its fastest mode, Kraken classifies 100 base pair reads at a rate of over 4.1 million reads per minute, 909 times faster than Megablast and 11 times faster than the abundance estimation program MetaPhlAn. Kraken is available at http://ccb.jhu.edu/software/kraken/.

## Background

Metagenomics, the study of genomic sequences obtained directly from an environment, has become an increasingly popular field of study in the past decade. In projects that have studied environments as varied as seawater [1], acidic mine drainage [2] and the human body [3], metagenomics has allowed researchers to create a picture of an environment’s microbial life without the need to isolate and culture individual microbes. Combined with an ability to sequence DNA quickly, metagenomics projects can generate a huge amount of sequence data that describes these previously invisible worlds.

For many metagenomic samples, the species, genera and even phyla present in the sample are largely unknown at the time of sequencing, and the goal of sequencing is to determine this microbial composition as precisely as possible. Of course, if an organism is completely unlike anything previously seen, then its DNA sequence cannot be characterized other than to label it as novel. Many species, though, have some detectable similarity to a known species, and this similarity can be detected by a sensitive alignment algorithm. The most well-known such algorithm, and one of the best methods for assigning a taxonomic label to an unknown sequence, is the BLAST program [4], which can classify a sequence by finding the best alignment to a large database of genomic sequences. Although BLAST was not designed for metagenomic sequences, it is easily adapted to this problem and it remains one of the best methods available [5].

Other methods of sequence classification have been proposed, utilizing sequence alignment and machine learning techniques in an attempt to improve upon BLAST’s accuracy. In the MEGAN [6] program, a sequence is searched (using BLAST) against multiple databases, and the lowest common ancestor (LCA) of the best matches against each database is assigned to the sequence. PhymmBL [5,7] combines the results of BLAST with scores produced from interpolated Markov models to a achieve higher accuracy than BLAST alone. The Naïve Bayes Classifier (NBC) [8] applies a Bayesian rule to distributions of *k*-mers within a genome. However, all these programs perform at speeds slower than BLAST, which itself takes very substantial CPU time to align the millions of sequences generated by a typical Illumina sequencing run. This processing burden is so demanding that it suggested another, faster approach to metagenomic sequence analysis: abundance estimation.

Abundance estimation programs work by creating a database that is much smaller than the collection of all genomes, which allows them to perform classification much faster than methods that attempt to identify every read in a data set. These databases are engineered to contain ‘marker’ genes (single-copy genes present in nearly all microbes) [9], or genes that have been found to be specific to certain clades [10]. Because the databases only contain a very small sample of each genome, these programs can only classify a small percentage of sequences from a typical metagenomics sample. They are meant to be used to characterize the distribution of organisms present in a given sample, rather than labelling every single read. For example, the initial analysis of the Human Microbiome Project [3] used one of these programs, MetaPhlAn [10], to analyze several trillion bases (terabases) of metagenomic sequences collected from hundreds of humans. Although abundance estimation programs provide a summary-level characterization of a metagenome, they cannot help with analyses that require more details about the sample. For example, they cannot be used to estimate the gene content in a sample because this requires every read to be compared to known genes. If a sample contains a large number of reads from one species, then it is sometimes possible to assemble those reads to reconstruct part or all of the genome [11], and then to classify the resulting contigs.

Here we describe Kraken, a new sequence classification tool whose accuracy is comparable to the best sequence classification techniques, and whose speed far exceeds both classifiers and abundance estimation programs. This speed advantage derives in large part from the use of exact-match database queries of *k*-mers, rather than inexact alignment of sequences. Its accuracy is made possible by the very large and still-growing number of sequenced microbial genomes, currently numbering over 8,500, which makes it likely that very similar sequences from a given species have been seen before. Through the use of a novel algorithm to process the disparate results returned by its database, Kraken is able to achieve genus-level sensitivity and precision that are very similar to that obtained by the fastest BLAST program, Megablast.

## Results and discussion

### *k*-mer to lowest common ancestor database

**Figure 1:**
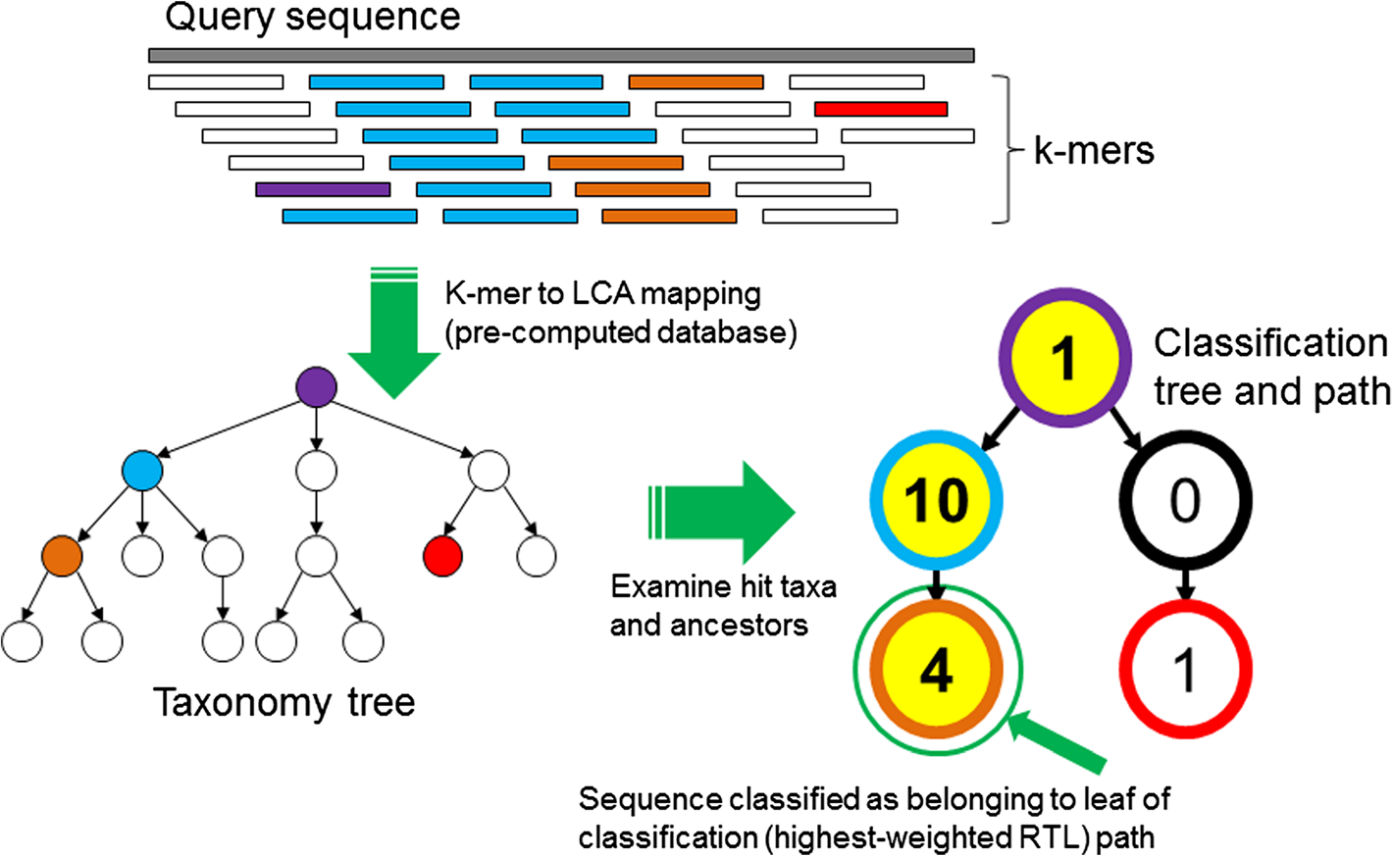
**The Kraken sequence classification algorithm.** To classify a sequence, each *k*-mer in the sequence is mapped to the lowest common ancestor (LCA) of the genomes that contain that *k*-mer in a database. The taxa associated with the sequence’s *k*-mers, as well as the taxa’s ancestors, form a pruned subtree of the general taxonomy tree, which is used for classification. In the classification tree, each node has a weight equal to the number of *k*-mers in the sequence associated with the node’s taxon. Each root-to-leaf (RTL) path in the classification tree is scored by adding all weights in the path, and the maximal RTL path in the classification tree is the classification path (nodes highlighted in yellow). The leaf of this classification path (the orange, leftmost leaf in the classification tree) is the classification used for the query sequence.

At the core of Kraken is a database that contains records consisting of a *k*-mer and the LCA of all organisms whose genomes contain that *k*-mer. This database, built using a user-specified library of genomes, allows a quick lookup of the most specific node in the taxonomic tree that is associated with a given *k*-mer. Sequences are classified by querying the database for each *k*-mer in a sequence, and then using the resulting set of LCA taxa to determine an appropriate label for the sequence (Figure 1 and Materials and methods). Sequences that have no *k*-mers in the database are left unclassified by Kraken. By default, Kraken builds the database with *k* = 31, but this value is user-modifiable.

### Simulated metagenome data

Although genuine metagenomic reads might provide the most realistic test of performance, such data would not allow us to assess classification accuracy, because the true species in metagenomic data sets today are mostly unknown. We instead used two simulated metagenomes created by combining real sequences obtained from projects that sequenced isolated microbial genomes. When creating these simulated metagenomes, we used data sequenced by the Illumina HiSeq and MiSeq sequencing platforms, and thus we call these the HiSeq and MiSeq metagenomes, respectively (see Materials and methods). These metagenomes were constructed to measure classification speed and genus-level accuracy for data generated by current and widely used sequencing platforms.

In addition to the two simulated metagenomes constructed with sequences from isolated genomes, we created a third metagenomic sample covering a much broader range of the sequenced phylogeny. This sample, featuring simulated bacterial and archaeal reads (called simBA-5), was created with an error rate five times higher than would be expected, to evaluate Kraken’s performance on data that contain many errors or have strong differences from Kraken’s genomic library (see Materials and methods).

### Classification accuracy

Classifiers generally adopt one of two strategies: for example, PhymmBL and NBC classify all sequences as accurately as possible, while Kraken and Megablast leave some sequences unclassified if insufficient evidence exists. Because PhymmBL and NBC label everything, they will tend to produce more false positives than methods like Kraken. In turn, one can expect a selective classifier to have higher precision at some cost to sensitivity. Uniquely among metagenomics classifiers, PhymmBL supplies confidence scores for its classifications, which can be used to discard low-confidence predictions and improve accuracy. Using a lower bound of 0.65 for genus-level confidence, we created a selective classifier based on PhymmBL’s predictions that we denote as PhymmBL65.

**Figure 2:**
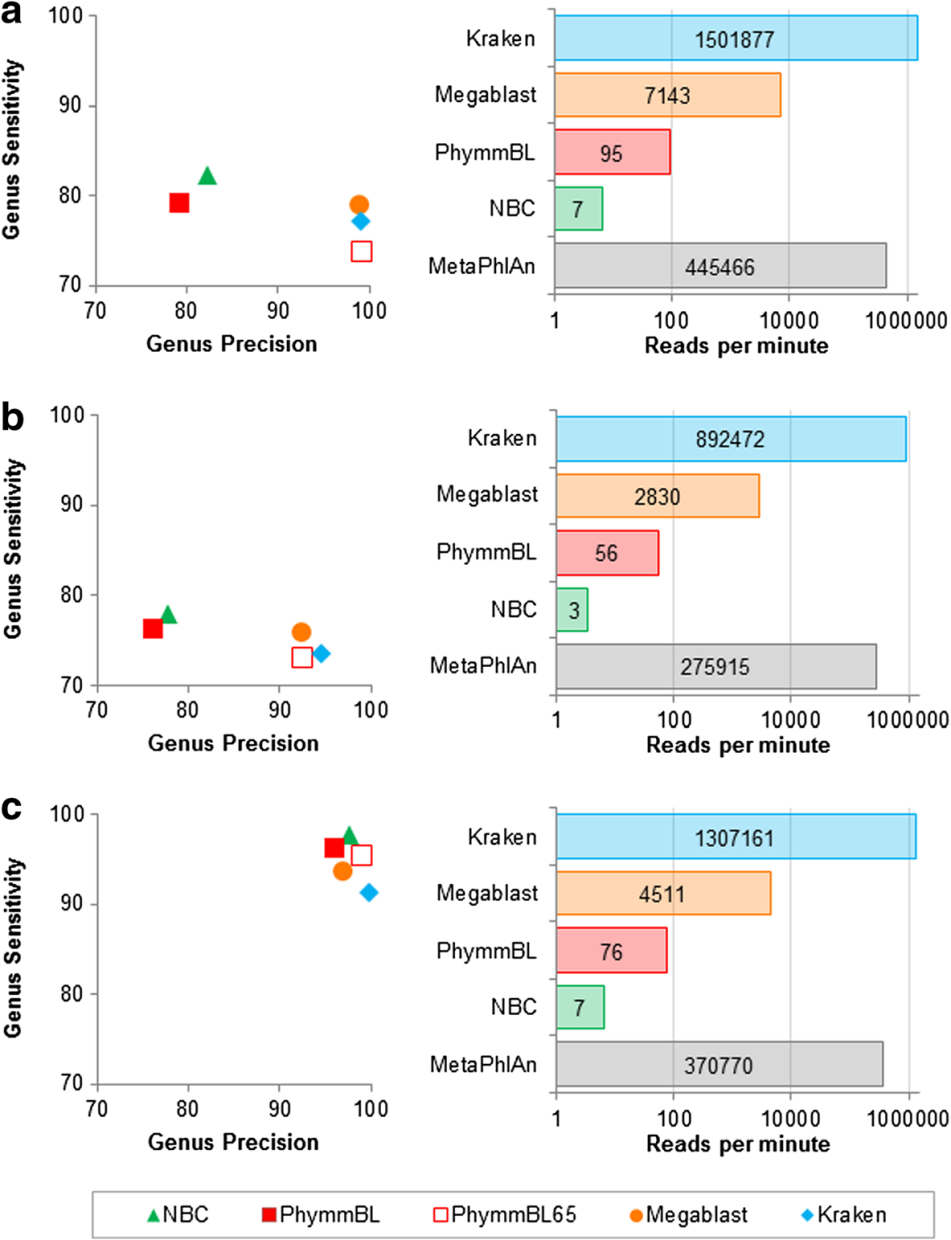
**Classification accuracy and speed comparison of classification programs for three simulated metagenomes.** For each metagenome, genus precision and sensitivity are shown for five classifiers, and speed is shown for five programs (PhymmBL65 is simply a confidence-filtered version of PhymmBL’s results, and MetaPhlAn only classifies a subset of reads that map to one of its marker genes, as it is an abundance estimation program). Results shown are for: **(a)** the HiSeq metagenome, consisting of HiSeq reads (mean length *μ* = 92 bp) in equal proportion from ten bacterial sequencing projects; **(b)** the MiSeq metagenome, consisting of MiSeq reads (*μ* = 156 bp) in equal proportion from ten bacterial projects; and **(c)** the simBA-5 metagenome, consisting of simulated 100-bp reads with a high error rate from 1,967 bacterial and archaeal taxa. Note that the horizontal axes in all speed graphs have a logarithmic scale.

To compare Kraken’s accuracy to these of other classification methods, we classified 10,000 sequences from each of our simulated metagenomes and measured genus-level sensitivity and precision (Figure 2 and Table 1). Here, sensitivity refers to the proportion of sequences assigned to the correct genus. Precision, also known as positive predictive value, refers to the proportion of correct classifications, out of the total number of classifications attempted. Kraken’s sensitivity and precision are very close to that of Megablast. For all three metagenomes, Kraken’s sensitivity was within 2.5 percentage points of Megablast’s. The use of exact 31-base matches, however, appears to yield a higher precision for Kraken, as its precision was the highest of all classifiers for each of the three metagenomes. As may be expected, the nonselective classifiers were able to achieve slightly higher sensitivity than the selective classifiers, but at the cost of a significantly lower precision, approximately 80% versus close to 100% for Kraken.

**Table 1:** Genus-level classification accuracy for three simulated metagenomes

	**HiSeq**		**MiSeq**		**simBA-5**	
**Classifier**	**Precision**	**Sensitivity**	**Precision**	**Sensitivity**	**Precision**	**Sensitivity**
Megablast	99.03	79.00	92.44	75.76	96.93	93.67
NBC	82.33	82.33	77.78	77.78	97.64	97.64
PhymmBL	79.14	79.14	76.21	76.21	96.11	96.11
PhymmBL65	99.13	73.95	92.47	73.03	99.08	95.45
Kraken	99.20	77.15	94.71	73.46	99.90	91.25
Kraken-Q	99.12	76.31	94.69	70.41	99.92	89.54
MiniKraken	99.44	66.12	97.41	67.95	99.95	65.87
MiniKraken-Q	99.36	65.67	97.32	65.84	99.98	65.31
Kraken-GB	99.51	93.75	98.48	86.23	99.48	91.13

We also note the recent publication of a method, LMAT [12], which uses a *k*-mer indexing scheme similar to Kraken’s, but otherwise differs in its classification strategy. LMAT cannot easily be downloaded and run on our simulated data (see Additional file 1: Note 1) so instead we ran Kraken on a data set used for LMAT’s published results. For that data (the PhymmBL set), Kraken exceeded LMAT’s accuracy in both identifying read origin and identifying the presence of species in the sample. Both methods had essentially perfect (near 100%) precision, but Kraken correctly labelled the species of 89% of the reads while LMAT only did so for 74% of the reads. However, as we note, that data set does not provide a good basis for comparison because the reads are simulated without error from genomes included in both Kraken’s and LMAT’s databases.

### Classification speed

Because of the very large size of metagenomic data sets today, classification speed is critically important, as demonstrated by the emergence of rapid abundance estimation programs such as MetaPhlAn. To evaluate classification speed, we ran each classifier, as well as MetaPhlAn, against each of the three metagenomes that we used to test accuracy (Figure 2).

Kraken classified reads much faster than any other classifier, with performance ranging from 150 to 240 times faster than the closest competitor. Kraken processed data at a rate of over 1.5 million reads per minute (rpm) for the HiSeq metagenome, over 1.3 million rpm for the simBA-5 metagenome and over 890,000 rpm for the MiSeq metagenome. The next fastest classifier, Megablast, had speeds of 7,143 rpm for the HiSeq metagenome, 4,511 rpm for the simBA-5 metagenome and 2,830 rpm for the MiSeq metagenome. For all three metagenomes, PhymmBL classified at a rate of <100 rpm and NBC at <10 rpm. Kraken is also more than three times as fast as MetaPhlAn (which only classifies a subset of reads), which had speeds of 445,000 rpm, 371,000 rpm and 276,000 rpm for the HiSeq, simBA-5 and MiSeq metagenomes, respectively. These results are shown in Figure 2. As expected, all tools processed the longer MiSeq reads (mean length *μ* = 156 bp) more slowly than the simBA-5 (*μ* = 100 bp) or HiSeq (*μ* = 92 bp) reads. We also performed a speed comparison against LMAT using one of the real samples discussed in LMAT’s published results; on this sample Kraken was 38.82 times faster than LMAT and 7.55 times faster than a version of LMAT using a smaller database (Additional file 1: Note 1).

### Other variants of Kraken

**Figure 3:**
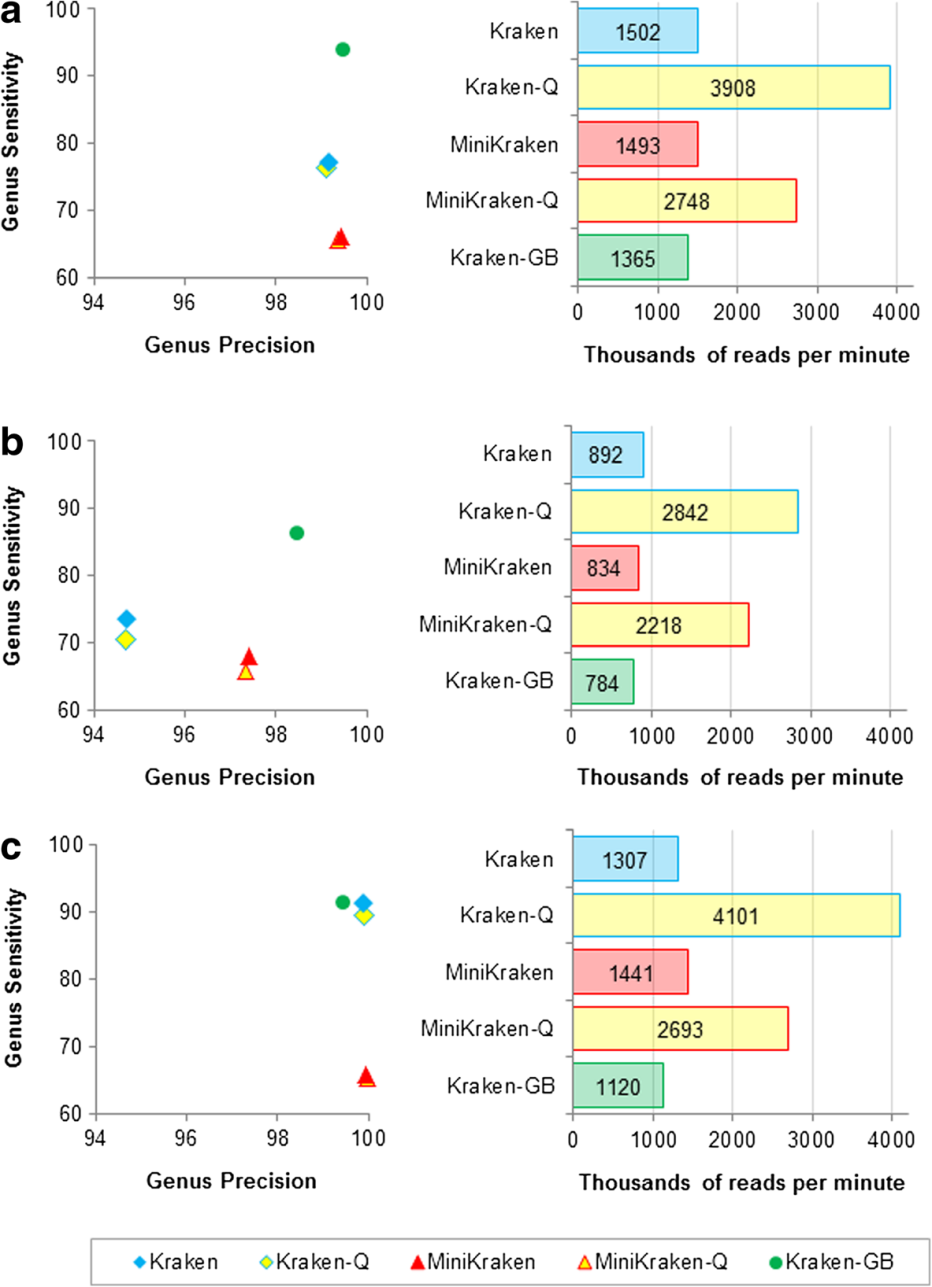
**Classification accuracy and speed comparison of variants of Kraken for three simulated metagenomes.** For each metagenome, genus precision and sensitivity are shown for five classifiers, and speed is shown for Kraken, along with a reduced memory version of Kraken (MiniKraken), quick execution versions of both (Kraken-Q and MiniKraken-Q), and Kraken run with a database containing draft and completed microbial genomes from GenBank (Kraken-GB). Results shown are for the same metagenomes used in Figure 2. Note that the scales of the axes differ from Figure 2, as the precision and speed of Kraken (and its variants) exceed that of the other classifiers used. **(a)** HiSeq metagenome. **(b)** MiSeq metagenome. **(c)** simBA-5 metagenome.

To obtain maximal speed, Kraken needs to avoid page faults (instances where data must be brought from a hard drive into physical memory), so it is important that Kraken runs on a computer with enough RAM to hold the entire database. Although Kraken’s default database requires 70 GB of RAM, we also developed a method to remove *k*-mers from the database, which dramatically reduces the memory requirements. We call this version of Kraken, which uses a smaller database, MiniKraken. For our results here, we used a 4 GB database. Compared to Kraken, the ability of MiniKraken to recognize species from short reads is lower, with sensitivity for our real sequence metagenomes dropping approximately 11% (Figure 3 and Table 1). On the high-error simBA-5 metagenome, MiniKraken’s sensitivity was more than 25 percentage points lower than Kraken’s, indicating that for short reads, high error rates can cause substantial loss in sensitivity. However, for all three metagenomes, MiniKraken was more precise than Kraken.

MiniKraken’s high precision demonstrates that in many cases we do not need to examine all *k*-mers in a sequence to get the correct classification. Taking this idea to its extreme, we developed a ‘quick operation’ mode for Kraken (and MiniKraken), where instead of querying all *k*-mers in a sequence against our database, we instead stop at the first *k*-mer that exists in the database, and use the LCA associated with that *k*-mer to classify the sequence. This operation mode (denoted by appending -Q to the classifier name) allows Kraken to skip tens or hundreds of *k*-mer queries per sequence, significantly increasing its classification speed with only a small fall in accuracy (Figure 3 and Table 1). Because a database containing fewer *k*-mers requires more queries from a sequence to find a hit, MiniKraken-Q is slower than Kraken-Q, even when MiniKraken is faster than Kraken.

We also created a variant Kraken database that contains GenBank’s draft and completed genomes for bacteria and archaea, which we call Kraken-GB. The regular version of Kraken only includes RefSeq complete genomes, of which there are 2,256, while Kraken-GB contains 8,517 genomes. Our hypothesis was that Kraken-GB would have a higher sensitivity than standard Kraken for our metagenomes, by virtue of its larger database. Kraken-GB has a much higher sensitivity for the HiSeq and MiSeq metagenomes compared to Kraken (Figure 3 and Table 1), primarily due to the presence of two genomes in these simulated metagenomic samples that have close relatives only in Kraken-GB’s database (Materials and methods).

Although Kraken-GB does have higher sensitivity than Kraken, it sometimes makes surprising errors, which we discovered were caused by contaminant and adapter sequences in the contigs of some draft genomes. These contaminant sequences come from other bacteria, viruses or even human genomes, and they result in incorrectly labelled *k*-mers in the database. We attempted to remove these from Kraken-GB (Materials and methods), but some contaminants may still slip through any filters. Thus for now, the default version of Kraken uses only complete RefSeq genomes.

### Clade exclusion experiments

An important goal of metagenomics is the discovery of new organisms, and the proper classification of novel organisms is a challenge for any classifier. Although a classifier cannot possibly give a novel species the proper species label, it may be able to identify the correct genus. To simulate the presence of novel organisms, we re-analyzed the simBA-5 metagenome after first removing organisms from the Kraken database that belonged to the same clade. That is, for each read, we masked out database hits for the species of the read’s origin, and evaluated Kraken’s accuracy at the higher ranks (e.g., genus and family). We continued this masking and evaluation process for clades of origin up to the phylum rank. This procedure approximates how Kraken would classify the metagenomic reads if that clade were not present in the database.

Table 2 contains the results of this analysis. Kraken exhibited high rank-level precision in all cases where a clade was excluded, with rank-level precision remaining at or above 93% for all pairs of measured and excluded ranks. However, sensitivity was dramatically lower: at best, Kraken was able to classify approximately 33% of reads when their species has never been seen before. This is not surprising in light of Kraken's reliance on exact matches of relatively long *k*-mers: sequences deriving from different genera rarely share long exact matches. Nonetheless, the high precision in this experiment indicates that when Kraken is presented with novel organisms, it is likely to either classify them properly at higher levels or not classify them at all.

**Table 2:** Classification statistics with clade exclusion for Kraken on the simBA-5 metagenome

**Measured rank**	**Excluded rank**					
	**Species**	**Genus**	**Family**	**Order**	**Class**	**Phylum**
Kingdom	100/24.4/24.4	100/7.9/7.9	100/2.8/2.8	100/2.3/2.3	100/1.5/1.5	100/1.1/1.1
Phylum	99.9/23.9/24.5	99.6/7.2/7.9	98.7/2.5/2.8	98.0/1.6/2.4	96.8/1.2/1.7	–
Class	99.7/24.7/25.3	99.1/7.1/7.9	96.7/2.0/3.0	93.2/1.0/2.3	–	–
Order	99.7/24.1/25.3	98.9/6.8/8.5	96.4/2.0/3.4	–	–	–
Family	99.7/25.4/26.7	98.5/8.5/10.8	–	–	–	–
Genus	99.2/26.3/33.2	–	–	–	–	–

For each measured rank, Kraken’s rank-level precision, sensitivity and classification percentage are shown. Classification percentage is defined here as the percentage of reads with taxonomic entries at both the measured and excluded ranks that were classified by Kraken with the clade of origin excluded.

### Human Microbiome Project data

**Figure 4:**
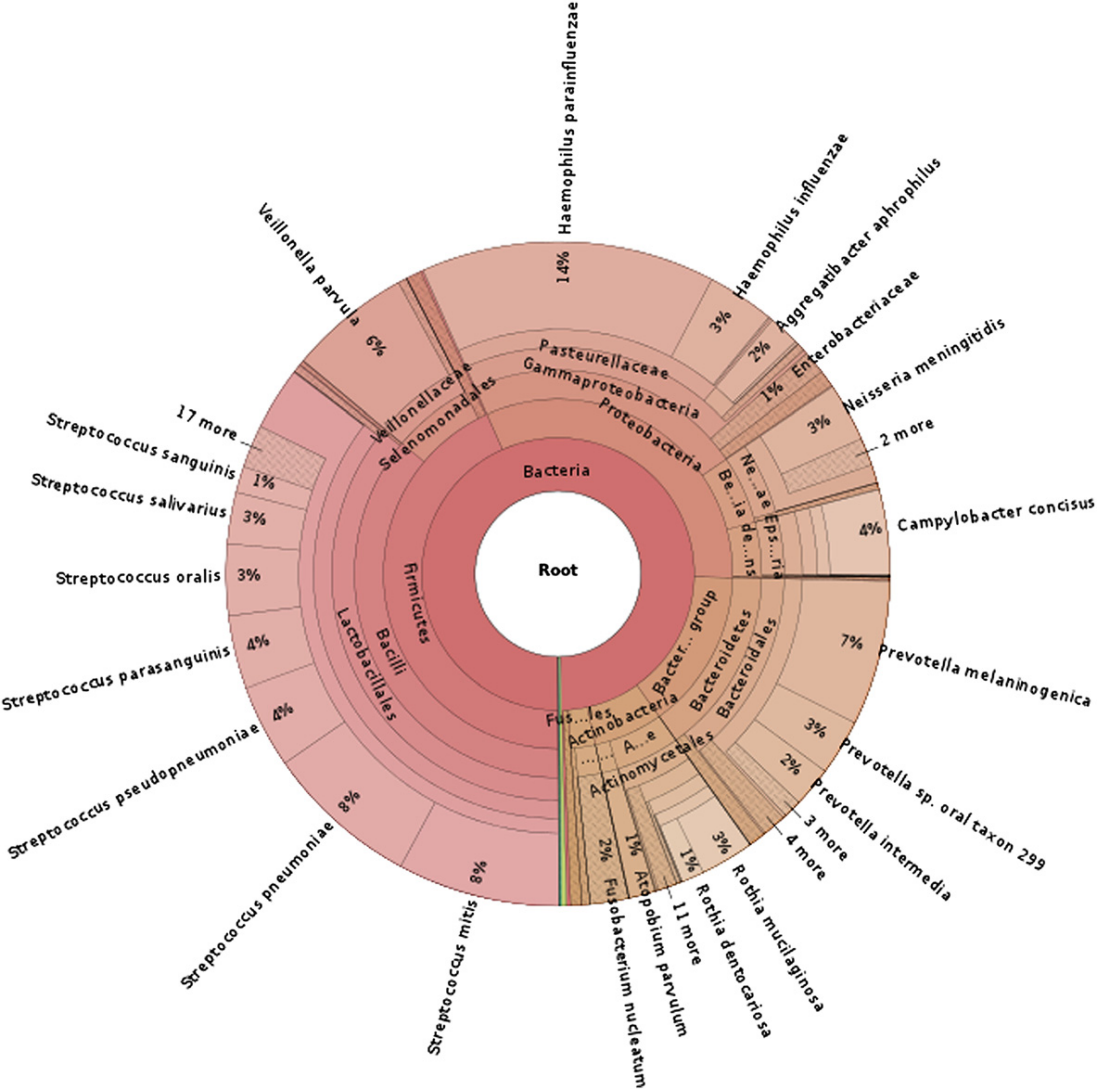
**Taxonomic distribution of saliva microbiome reads classified by Kraken.** Sequences from saliva samples collected from three individuals were classified by Kraken. The distribution of those reads that were classified by Kraken is shown.

We used Kraken to classify reads from three saliva samples collected as part of the Human Microbiome Project. Because these samples were obtained from humans, we created a Kraken database containing bacterial, viral and human genomes to classify these reads. Combining the three samples together, we report the taxonomic distribution of the classified reads (Figure 4). An analysis of the classified reads from the combined samples reveals that a majority of those reads were classified into one of three genera: *Streptococcus* (30%), *Haemophilus* (17%) and *Prevotella* (13%). *Streptococcus mitis*[13], *Haemophilus parainfluenzae*[14] and *Prevotella melaninogenica*[15], the most abundant species (by read count) of each of these three genera, are all known to be associated with human saliva. We also performed the classification on each sample separately (Additional file 1: Figures S1,S2,S3).

Of note is that 68.2% of the reads were not classified by Kraken. To determine why these reads were not classified by Kraken, we aligned a randomly selected subset of 2,500 of these unclassified reads to the RefSeq bacterial genomes using BLASTN. Only 11% (275) of the subset of unclassified reads had a BLASTN alignment with E-value ≤ 10^−5^ and identity ≥90%. This suggests that the vast majority of the reads not classified by Kraken were significantly different from any known species, and thus simply impossible to identify.

## Conclusions

Kraken’s accuracy is comparable to that of Megablast for classifying short sequence reads, as might be expected given that both require long exact sequence matches (Kraken requires 31 bp exact matches, while Megablast requires 28 bp [16]). As we showed for the simBA-5 metagenome, where high sequence error rates were simulated, Megablast’s inexact alignment strategy allowed it to tolerate more errors and achieve higher sensitivity than Kraken, which uses only exact alignment. We note that even in the face of this high error rate, Kraken’s sensitivity still exceeded 90% and its precision was 99.9%. With Kraken’s high precision, users concerned with maximizing sensitivity could run Kraken first, and then run another classification program on the reads not classified by Kraken, obtaining high sensitivity results much faster than with a single program.

An important constraint for Kraken is its memory usage: at present, the default database requires 70 GB, a value that will grow in linear proportion to the number of distinct *k*-mers in the genomic library (the database’s records occupy 12 bytes per *k*-mer). For comparison, the only other *k*-mer-based classifier, LMAT, uses a far larger database of 619 GB. While Kraken only stores the LCA for each *k*-mer, LMAT also records all genomes associated with a *k*-mer, resulting in a record size bounded only by the number of genomes in the library. The use of a reduced database by MiniKraken offers a nearly equivalent alternative, if Kraken’s database is too large for the available computational resources.

One important potential alternative use of Kraken is to identify contaminant sequences rapidly. As we noted, some of the draft microbial genomes in GenBank contain contaminating sequences from many different sources. A fast classifier like Kraken can quickly identify many such contaminants before they are included in a draft assembly. Similarly, for microbial samples collected from humans, a Kraken database can be created, which can be used to identify contaminating human reads in a metagenomic sample quickly.

Finally, Kraken’s results demonstrate the high speed and accuracy that are achievable through the use of short exact alignments. The Kraken database structure, which is tuned to query overlapping *k*-mers rapidly, enables Kraken to produce results faster than would be possible without the database facilitating this type of query. We believe that this structure can find a use in other applications beyond taxonomic classification; for example, de Bruijn graphs, commonly used in genome assembly programs, can effectively be traversed by querying a database with overlapping *k*-mers [17], and that process can be made faster through the caching behavior of the Kraken database. Likewise, most operations that need to query overlapping *k*-mers should be able to run significantly faster by using a data structure like the Kraken database.

## Materials and methods

### Sequence classification algorithm

To classify a DNA sequence *S*, we collect all *k*-mers within that sequence into a set, denoted as *K*(*S*). We then map each *k*-mer in *K*(*S*), using the algorithm described below, to the LCA taxon of all genomes that contain that *k*-mer. These LCA taxa and their ancestors in the taxonomy tree form what we term the *classification tree*, a pruned subtree that is used to classify *S*. Each node in the classification tree is weighted with the number of *k*-mers in *K*(*S*) that mapped to the taxon associated with that node. Then, each root-to-leaf (RTL) path in the classification tree is scored by calculating the sum of all node weights along the path. The maximum scoring RTL path in the classification tree is the *classification path*, and *S* is assigned the label corresponding to its leaf (if there are multiple maximally scoring paths, the LCA of all those paths’ leaves is selected). This algorithm, illustrated in Figure 1, allows Kraken to consider each *k*-mer within a sequence as a separate piece of evidence, and then attempt to resolve any conflicting evidence if necessary. Note that for an appropriate choice of *k*, most *k*-mers will map uniquely to a single species, greatly simplifying the classification process. Sequences for which none of the *k*-mers in *K*(*S*) are found in any genome are left unclassified by this algorithm.

The use of RTL path scoring in the classification tree is necessary in light of the inevitable differences between the sequences to be classified and the sequences present in any library of genomes. Such differences can, even for large values of *k*, result in a *k*-mer that is present in the library but associated with a species far removed from the true source species. By scoring the various RTL paths in the classification tree, we can compensate for these differences and correctly classify sequences even when a small minority of *k*-mers in a sequence indicate that the sequence should be assigned an incorrect taxonomic label.

### Database creation

Efficient implementation of Kraken’s classification algorithm requires that the mapping of *k*-mers to taxa is performed by querying a pre-computed database. Kraken creates this database through a multi-step process, beginning with the selection of a library of genomic sequences. Kraken includes a default library, based on completed microbial genomes in the National Center for Biotechnology Information’s (NCBI) RefSeq database, but the library can be customized as needed by individual users [18].

Once the library is chosen, we use the Jellyfish multithreaded *k*-mer counter [19] to create a database containing every distinct 31-mer in the library. Once the database is complete, the 4-byte spaces Jellyfish used to store the *k*-mer counts in the database file are instead used by Kraken to store the taxonomic ID numbers of the *k*-mers’ LCA values. After the database has been created by Jellyfish, the genomic sequences in the library are processed one at a time. For each sequence, the taxon associated with it is used to set the stored LCA values of all *k*-mers in the sequence. As sequences are processed, if a *k*-mer from a sequence has had its LCA value previously set, then the LCA of the stored value and the current sequence’s taxon is calculated and that LCA is stored for the *k*-mer. Taxon information is obtained from the NCBI taxonomy database.

### Database structure and search algorithm

Because Kraken very frequently uses a *k*-mer as a database query immediately after querying an adjacent *k*-mer, and because adjacent *k*-mers share a substantial amount of sequence, we utilize the minimizer concept [20] to group similar *k*-mers together. To explain our application of this concept, we here define the canonical representation of a DNA sequence *S* as the lexicographically smaller of *S* and the reverse complement of *S*. To determine a *k*-mer’s minimizer of length *M*, we consider the canonical representation of all *M*-mers in the *k*-mer, and select the lexicographically smallest of those *M*-mers as the *k*-mer’s minimizer. In practice, adjacent *k*-mers will often have the same minimizer.

**Figure 5:**
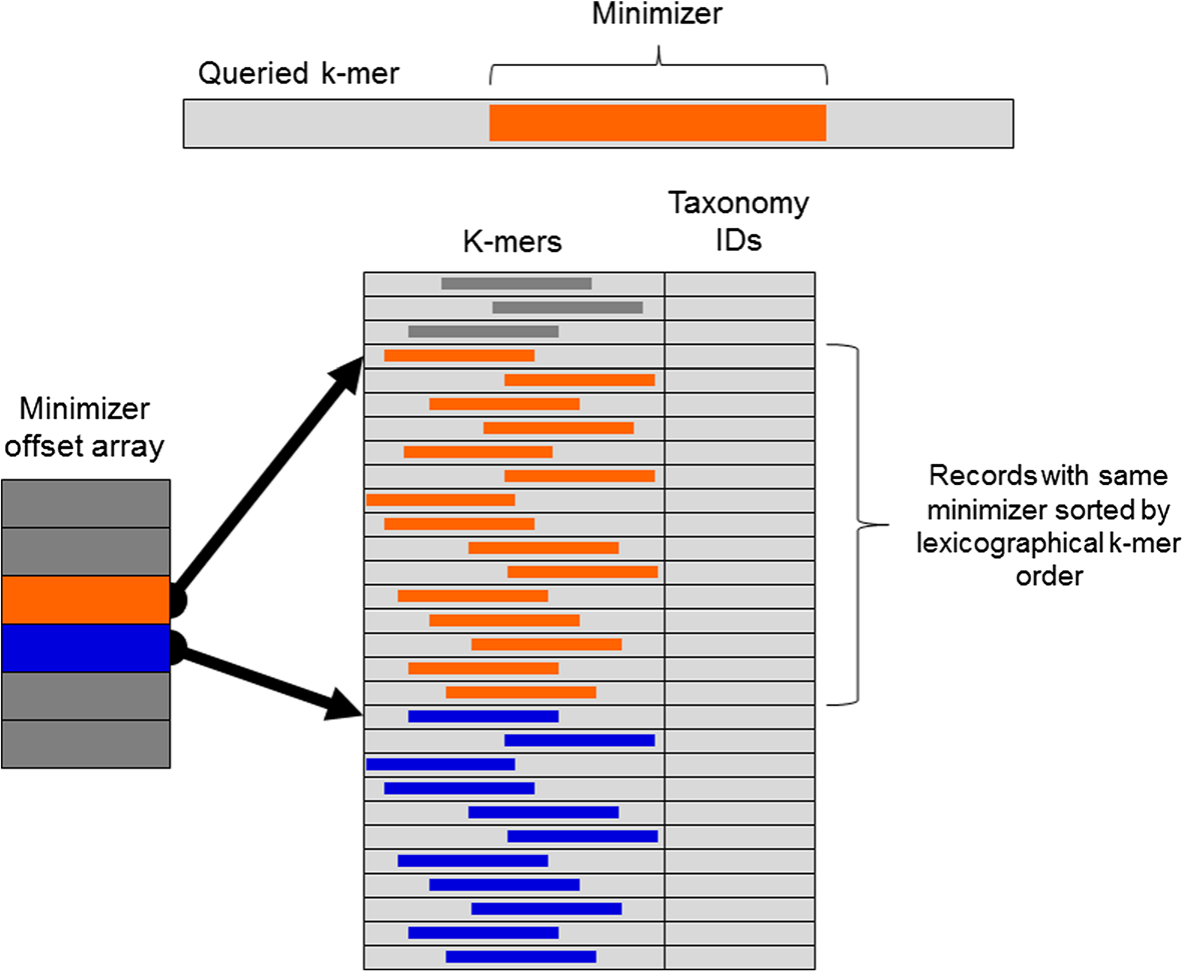
**Kraken database structure.** Each *k*-mer to be queried against the database has a specific substring that is its minimizer. To search for a *k*-mer in the database, the positions in the database that contain *k*-mers with the same minimizer are examined. These positions are quickly found by examining the minimizer offset array for the start positions of records with the *k*-mer’s minimizer (orange) and the next possible minimizer (blue). Within a range of records associated with a given minimizer, records are sorted by lexicographical ordering of their *k*-mers, allowing a query to be completed by using a binary search over this range.

In Kraken’s database, all *k*-mers with the same minimizer are stored consecutively, and are sorted in lexicographical order of their canonical representations. A query for a *k*-mer *R* can then be processed by looking up in an index the positions in the database where the *k*-mers with *R*’s minimizer would be stored, and then performing a binary search within that region (Figure 5). Because adjacent *k*-mers often have the same minimizer, the search range is often the same between two consecutive queries, and the search in the first query can often bring data into the CPU cache that will be used in the second query. By allowing memory accesses in subsequent queries to access data in the CPU cache instead of RAM, this strategy makes subsequent queries much faster than they would otherwise be. The index containing the offsets of each group of *k*-mers in the database requires 8 × 4^*M*^ bytes. By default Kraken uses 15-bp minimizers, but the user can modify this value; for example, in creating MiniKraken, we used 13-bp minimizers to ensure the total database size stayed under 4 GB.

In implementing Kraken, we made further optimizations to the structure and search algorithm described above. First, as noted by Roberts *et al*. [20], a simple lexicographical ordering of *M*-mers can result in a skewed distribution of minimizers that over-represents low-complexity *M*-mers. In Kraken, such a bias would create many large search ranges, which would require more time to search. To create a more even distribution of minimizers (and thus speed up searches), we use the exclusive-or (XOR) operation to toggle half of the bits of each *M*-mer’s canonical representation prior to comparing the *M*-mers to each other using lexicographical ordering. This XOR operation effectively scrambles the standard ordering, and prevents the large bias toward low-complexity minimizers.

We also take advantage of the fact that the search range is often the same between queries to make Kraken’s queries faster. Rather than compute the minimizer each time we perform a query, we first search the previous range. If our queried *k*-mer is found in this range, the query can return immediately. If the *k*-mer is not found, then the minimizer is computed; if the *k*-mer’s minimizer is the same as the last queried *k*-mer’s, then the query fails, as the minimizer’s search space has been shown not to have the *k*-mer. Only if the minimizer has changed does Kraken have to adjust the search range and search again for the *k*-mer.

### Constructing simulated metagenomes

The HiSeq and MiSeq metagenomes were built using 20 sets of bacterial whole-genome shotgun reads. These reads were found either as part of the GAGE-B project [21] or in the NCBI Sequence Read Archive. Each metagenome contains sequences from ten genomes (Additional file 1: Table S1). For both the 10,000 and 10 million read samples of each of these metagenomes, 10% of their sequences were selected from each of the ten component genome data sets (i.e., each genome had equal sequence abundance). All sequences were trimmed to remove low quality bases and adapter sequences.

The composition of these two metagenomes poses certain challenges to our classifiers. For example, *Pelosinus fermentans*, found in our HiSeq metagenome, cannot be correctly identified at the genus level by Kraken (or any of the other previously described classifiers), because there are no *Pelosinus* genomes in the RefSeq complete genomes database; however, there are seven such genomes in Kraken-GB’s database, including six strains of *P. fermentans*. Similarly, in our MiSeq metagenome, *Proteus vulgaris* is often classified incorrectly at the genus level because the only *Proteus* genome in Kraken’s database is a single *Proteus mirabilis* genome. Five more *Proteus* genomes are present in Kraken-GB’s database, allowing Kraken-GB to classify reads better from that genus. In addition, the MiSeq metagenome contains five genomes from the Enterobacteriaceae family (*Citrobacter*, *Enterobacter*, *Klebsiella*, *Proteus* and *Salmonella*). The high sequence similarity between the genera in this family can make distinguishing between genera difficult for any classifier.

The simBA-5 metagenome was created by simulating reads from the set of complete bacterial and archaeal genomes in RefSeq. Replicons from those genomes were used if they were associated with a taxon that had an entry associated with the genus rank, resulting in a set of replicons from 607 genera. We then used the Mason read simulator [22] with its Illumina model to produce 10 million 100-bp reads from these genomes. First we created simulated genomes for each species, using a SNP rate of 0.1% and an indel rate of 0.1% (both default parameters), from which we generated the reads. For the simulated reads, we multiplied the default mismatch and indel rates by five, resulting in an average mismatch rate of 2% (ranging from 1% at the beginning of reads to 6% at the ends) and an indel rate of 1% (0.5% insertion probability and 0.5% deletion probability). For the simBA-5 metagenome, the 10,000 read set was generated from a random sample of the 10 million read set.

### Evaluation of accuracy and speed

We elected to measure accuracy primarily at the genus level, which was the lowest level for which we could easily determine the taxonomy information for PhymmBL and NBC’s predictions in an automated fashion. (This is due to the manner in which PhymmBL and NBC report their results). Because some genomes do not have taxonomic entries at all seven ranks (species, genus, family, order, class, phylum and kingdom), we defined genus-level sensitivity as *A*/*B*, where *A* is the number of reads with an assigned genus that were correctly classified at that rank, and *B* is the total number of reads with any assigned genus. We defined sensitivity similarly for other taxonomic ranks.

Because Kraken may classify a read at levels above the species, measuring its precision requires us to define the effect on precision of assigning the correct genus (for example) while not assigning a species at all. For this reason, we defined rank-level precision as *C*/(*D* + *E*), where *C* is the number of reads labeled at or below the correct taxon at the measured rank, *D* is the number of reads labeled at or below the measured rank, and *E* is the number of reads incorrectly labeled above the measured rank. For example, given a read *R* that should be labeled as *Escherichia coli*, a labeling of *R* as *E. coli*, *E. fergusonii* or *Escherichia* would improve genus-level precision. A label of Enterobacteriaceae (correct family) or Proteobacteria (correct phylum) would have no effect on genus-level precision. A label for *R* of *Bacillus* (incorrect genus) or Firmicutes (incorrect phylum) would decrease the genus-level precision.

When evaluating PhymmBL’s accuracy, following its developers’ advice [7], we selected a genus confidence threshold for our comparisons. We selected 3,333 reads from the simulated medium complexity (simMC) [23] data set, covering 31 different genera. To simulate short reads from the Sanger sequence data in the simMC set, we selected the last 100 bp from each of the reads. We then ran PhymmBL against those 100-bp reads, and evaluated the genus-level sensitivity and precision of PhymmBL’s predictions with genus confidence thresholds from 0 to 1, in increments of 0.05. We found that a threshold of 0.65 yielded the highest *F*-score (the harmonic mean of sensitivity and precision), with 0.60 and 0.70 also having *F*-scores within 0.5 percentage points of the maximum (Additional file 1: Table S2). We therefore used the 0.65 genus confidence threshold in our comparisons. Although the selection of a threshold depends on a user’s individual needs, and so is to some extent arbitrary, a threshold selected in this manner provides a more proper comparison to a selective classifier such as Kraken than no threshold at all.

The time and accuracy results when using Megablast as a classifier were obtained from the log data produced by PhymmBL, as PhymmBL uses Megablast for its alignment step. When assigning a taxonomic label to a read with Megablast, we used the taxon associated with the first reported alignment. Megablast was run with default options.

Speed was evaluated using the single-threaded operation of each program (except for NBC). PhymmBL was altered so that its call to the blastn program used one thread instead of two. NBC was run with 36 concurrent processes operating on disjoint sets of genomes in its genomic library, and the total time for the classifier was determined by summing the decompression and scoring times for each genome. Wall clock times were recorded for all classifiers. In comparing Kraken to the other classifiers, we used BLAST+ 2.2.27, PhymmBL 4.0, NBC 1.1 and MetaPhlAn 1.7.6. Classifiers were all run on the same computer, with 48 AMD Opteron 6172 2.1 GHz CPUs and 252 GB of RAM, running Red Hat Enterprise Linux 5. The data sets used for speed evaluation had 10,000 reads each for all programs other than Kraken (and its variants) and MetaPhlAn, which used 10,000,000 read data sets. Higher read numbers were used with these faster programs to minimize the effect of the initial and final operations that take place during the programs’ execution.

Although Kraken is the only one of the programs we examined that explicitly performs operations to ensure its data is in physical memory before classification, we wanted to be sure that all programs were evaluated in a similar manner. When evaluating speed, for each program, we read all database files (e.g. IMM files and BLAST databases for PhymmBL, *k*-mer frequency lists for NBC and the Bowtie index for MetaPhlAn) into memory three times before running the program, in order to place the database content in the operating system cache (which is stored in physical memory).

### Reduced database sizes

To generate the 4-GB database for our MiniKraken results, we removed the first 18 of every block of 19 records in the standard Kraken database. A shrinking factor of 19 was selected as it was the smallest integer factor that would reduce the size to less than 4 GB, a size that can easily fit into the memory of many common personal computers. For users that have more RAM available, Kraken allows a smaller shrinking factor to be used, which will give increased sensitivity.

### Use of draft genomes

When constructing the Kraken-GB database, we noticed there were several contigs with known adapter sequences at the ends. In subsequent tests, we also found that some sequences in samples with large amounts of human sequence were consistently misclassified by this database, leading us to conclude that contamination was likely present in the draft genomes. In an attempt to counteract this contamination, we removed from the database those *k*-mers from known adapter sequences, as well as the first and last 20 *k*-mers from each of the draft contigs. While this did improve classification, it did not eliminate the misclassification problem. For this reason, we believe that if draft genomes are used in a Kraken database, very stringent measures should be used to remove contaminant sequences from the genomic library.

### Clade exclusion experiments

When re-analyzing the simBA-5 data set for our clade exclusion experiments, some reads were not used for certain pairs of measured and excluded ranks. If a read’s origin lacked a taxonomic entry at either of the measured or excluded ranks, it was not used for that particular experiment.

In addition, a read was not used in an experiment unless at least two other taxa represented in our database (aside from the excluded clade) at the excluded rank shared the clade of origin’s taxon at the measured rank. For example, a read from genus *G* would not be used in an experiment measuring accuracy at the class rank and excluding the genus rank unless *G*’s home class had at least two other genera with genomes in Kraken’s genomic library. Without this filtering step, were a genus excluded when it was the only genus in its class, Kraken could not possibly name the correct class, as all entries in the database from that class would be excluded as well. This is the same approach taken in similar experiments that were used to evaluate PhymmBL [5].

### Human microbiome data classification

We classified the Human Microbiome Project data using a Kraken database made from complete RefSeq bacterial, archaeal and viral genomes, along with the GRCh37 human genome. We retrieved the sequences of three accessions (SRS019120, SRS014468 and SRS015055) from the NCBI Sequence Read Archive, and each accession had two runs submitted. All reads were trimmed to remove low quality bases and adapter sequences. Krona [24] was used to generate all taxonomic distribution plots.

Because the sequences were all paired reads, we joined the reads together by concatenating the mates with a sequence of ‘NNNNN’ between them. Kraken ignores *k*-mers with ambiguous nucleotides, so the *k*-mers that span these ‘N’ characters do not affect classification. This operation allowed Kraken to classify a pair of reads as a single unit rather than having to classify the mates separately.

### Software and data availability

Kraken is written in C++ and Perl, and is available for download at [25] along with the metagenome data used to evaluate the accuracy of the classifiers presented here, and a downloadable 4-GB MiniKraken database similar to the one used here. The source code is also available from GitHub [26].

## Abbreviations

bp: base pair; CPU: central processing unit; LCA: lowest common ancestor; NBC: Naïve Bayes Classifier; NCBI: National Center for Biotechnology Information; rpm: reads per minute; RTL: root-to-leaf; simMC: simulated medium complexity; SNP: single nucleotide polymorphism.

## Competing interests

The authors declare that they have no competing interests.

## Authors’ contributions

DEW wrote the software and performed the experiments and analysis. DEW and SLS designed the experiments and wrote the paper. Both authors read and approved the final manuscript.

## Supplementary Material

Additional file 1 Supplementary information. Click here for file
