# Supplementary material for: Kraken: ultrafast metagenomic sequence classification using exact alignments
Source: Genome Biol. 2014 Mar 3;15(3):R46. doi: 10.1186/gb-2014-15-3-r46 (PMC4053813; doi:10.1186/gb-2014-15-3-r46)
Supplement: Additional file 1 — Supplementary information. [file gb-2014-15-3-r46-S1.pdf]

Supplementary Information

Supplementary Note 1

As LMAT requires over 4,300 CPU hours to build its database [12], over half a terabyte of RAM, and superuser privileges to use its memory allocation procedures, we were unable to create a local installation and run LMAT against the three metagenomes we developed to evaluate Kraken. Instead, we have run Kraken against some of the data used in LMAT's published results, and report our comparisons here.

LMAT has the ability to be run with two databases: kFull, the complete database, and kML, a database containing only "marker" k-mers that are the most "taxonomically informative" k-mers in the complete database. kFull (using k = 20) is 619 GB in size, while kML (using k = 18) is 39 GB. The authors reported results using both databases. As the relationship between LMAT-kFull and LMAT-kML is somewhat analogous to that between Kraken and MiniKraken, we report both Kraken and MiniKraken in our comparisons to LMAT.

The PhymmBL dataset used in evaluating LMAT's accuracy was formed by extracting 50 simulated 100 bp reads from each replicon that existed in RefSeq's set of completed bacterial and archaeal genomes as of October 2008[5]. The simBA-5 metagenome is similar to this dataset, but the addition of simulated error is a crucial difference, as the PhymmBL dataset was simulated without error. Because both LMAT and Kraken used RefSeq genomes to build their k-mer database, and the sequences in the PhymmBL dataset were drawn without modification from that library, both classifiers should achieve very high accuracy because they are being tested on data contained in their training sets. Nonetheless, as it was the only dataset to which we have access and for which we have LMAT's classification results, we use it here, and report LMAT's published results on this dataset.

The first experiment was to classify the dataset, which contained 540 distinct species, and report the number of species correctly identified in the dataset. Kraken identified 538 species, and MiniKraken identified 536, with neither mistakenly declaring the presence of a species not in the dataset. LMAT-kFull and LMAT-kML also had no false declarations of species presence, but only identified 531 and 527 species, respectively.

In the second experiment, we examined the individual reads to determine if they were classified at the species level, and if so, if they were correctly classified. Neither Kraken nor MiniKraken erroneously classified the species of any read. Kraken correctly identified the species of 88.68% of the reads, and MiniKraken correctly identified 85.06% of the reads' species. LMAT-kFull correctly identified the species of 74.2% of the reads, with 99.8% of species-level classifications being correct. LMAT-kML correctly identified the species of 40.4% of the reads, with 99.7% of species-level classifications being correct.

Since we do not have a local installation of LMAT, we cannot report LMAT's speed on any of our metagenomes. However, LMAT has published speeds for a human microbiome metagenome (SRA ERR011121), consisting of 33,123,975 75 bp reads. LMAT's raw speeds are reported in Kbp/s, but are the

result of 40-threaded execution; we therefore divide their reported speeds by 40 here. LMAT-kFull classified the sample at a speed of 63.7 Kbp/s on a single core, and LMAT-kML classified the sample at a speed of 327.4 Kbp/s on a single core. We downloaded this sample and classified it using Kraken and MiniKraken, using a single thread. Kraken classified the sample in 1005 seconds, for a classification speed of 2473 Kbp/s; MiniKraken took 915 seconds, for a speed of 2714 Kbp/s.

Supplementary Tables

Table S1: Component genomes in the HiSeq and MiSeq simulated metagenomes

Metagenome	Genome	Source
HiSeq	*Aeromonas hydrophila* SSU	GAGE-B web site
HiSeq	*Bacillus cereus* VD118	GAGE-B web site
HiSeq	*Bacteroides fragilis* HMW615	GAGE-B web site
HiSeq	*Mycobacterium abscessus* 6G-0125-R	GAGE-B web site
HiSeq	*Pelosinus fermentans* A11	SRA run SRR515982
HiSeq	*Rhodobacter sphaeroides* 2.4.1	GAGE-B web site
HiSeq	*Staphylococcus aureus* M0927	GAGE-B web site
HiSeq	*Streptococcus pneumoniae* TIGR4	SRA run SRR387337
HiSeq	*Vibrio cholerae* CP1032(5)	GAGE-B web site
HiSeq	*Xanthomonas axonopodis pv. Manihotis* UA323	GAGE-B web site
MiSeq	*Bacillus cereus* VD118	GAGE-B web site
MiSeq	*Citrobacter freundii* 47N	SRA run SRR493656
MiSeq	*Enterobacter cloacae*	SRA run SRR568037
MiSeq	*Klebsiella pneumoniae* NES14	SRA run SRR493683
MiSeq	*Mycobacterium abscessus* 6G-0125-R	GAGE-B web site
MiSeq	*Proteus vulgaris* 66N	SRA run SRR493654
MiSeq	*Rhodobacter sphaeroides* 2.4.1	GAGE-B web site
MiSeq	*Staphylococcus aureus* ST22	SRA run ERR103400
MiSeq	*Salmonella enterica* Montevideo str. N19965	SRA run SRR387337
MiSeq	*Vibrio cholerae* CP1032(5)	GAGE-B web site

Some data were obtained from the GAGE-B project web site (http://ccb.jhu.edu/gage_b/), while others were found through searches of the NCBI Sequence Read Archive (SRA).

Table S2: PhymmBL classification accuracy across different confidence levels

Confidence Level	Accuracy	Precision	Sensitivity
0.0	54.9	54.9	54.9
	(results equal for all confidence levels from 0-0.45)		
0.45	54.9	54.9	54.9
0.50	55.2	55.5	54.9
0.55	66.8	86.1	54.5
0.60	69.6	97.5	54.2
0.65	69.7	98.0	54.1
0.70	69.5	98.2	53.8
0.75	68.6	98.2	52.7
0.80	61.8	98.1	45.1
0.85	26.4	97.7	15.3
0.90	0.1	100.0	0.0
0.95	No labels at or above this confidence level		

Genus-level accuracy for PhymmBL on 3,333 reads from the simMC dataset is shown, with varying genus confidence thresholds applied. Note that when only labels with genus confidence >= 0.9 were considered, only 1 label remained.

Supplementary Figures

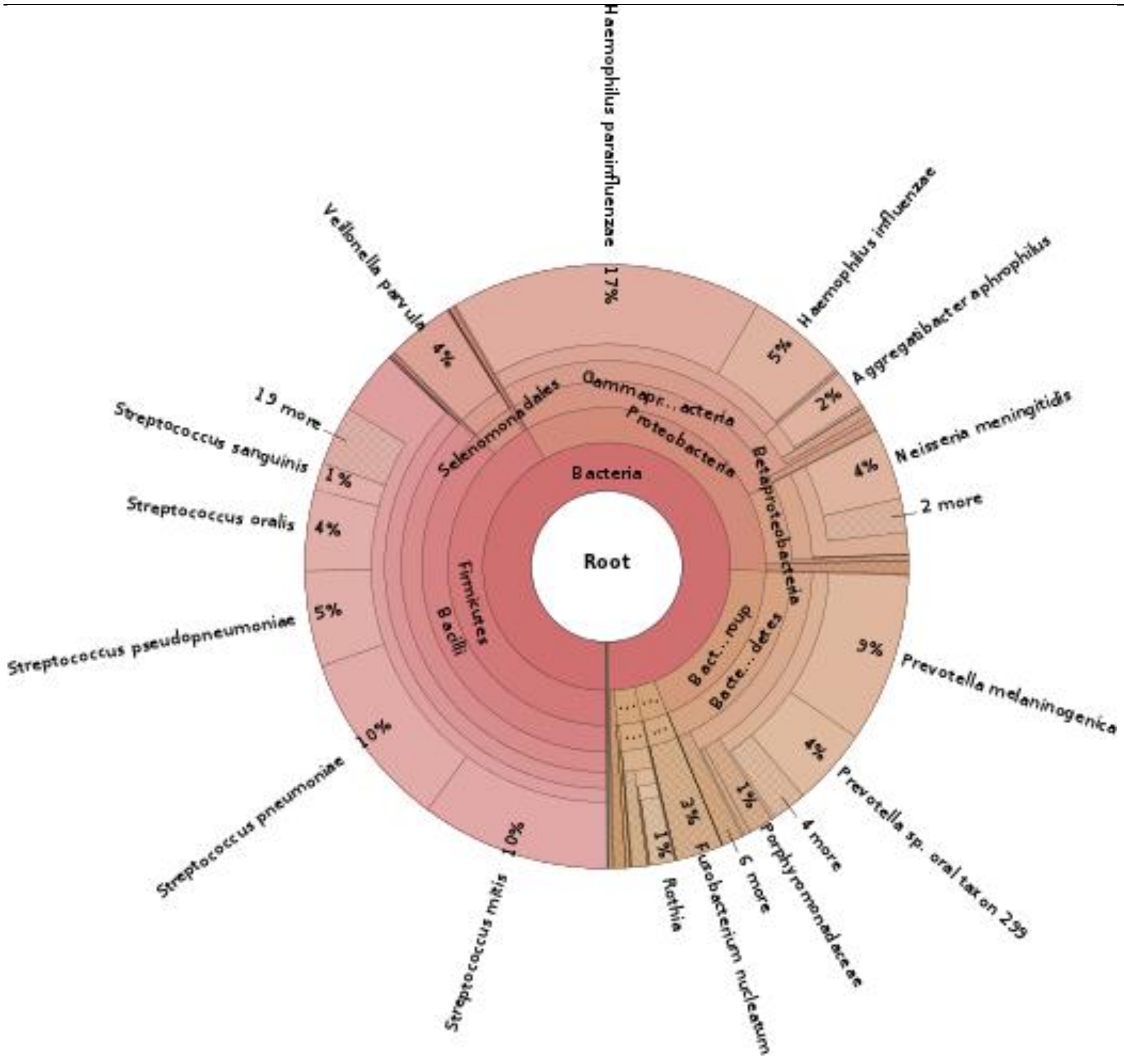

Figure S1: Taxonomic distribution of classified reads from SRA accession SRS019120.

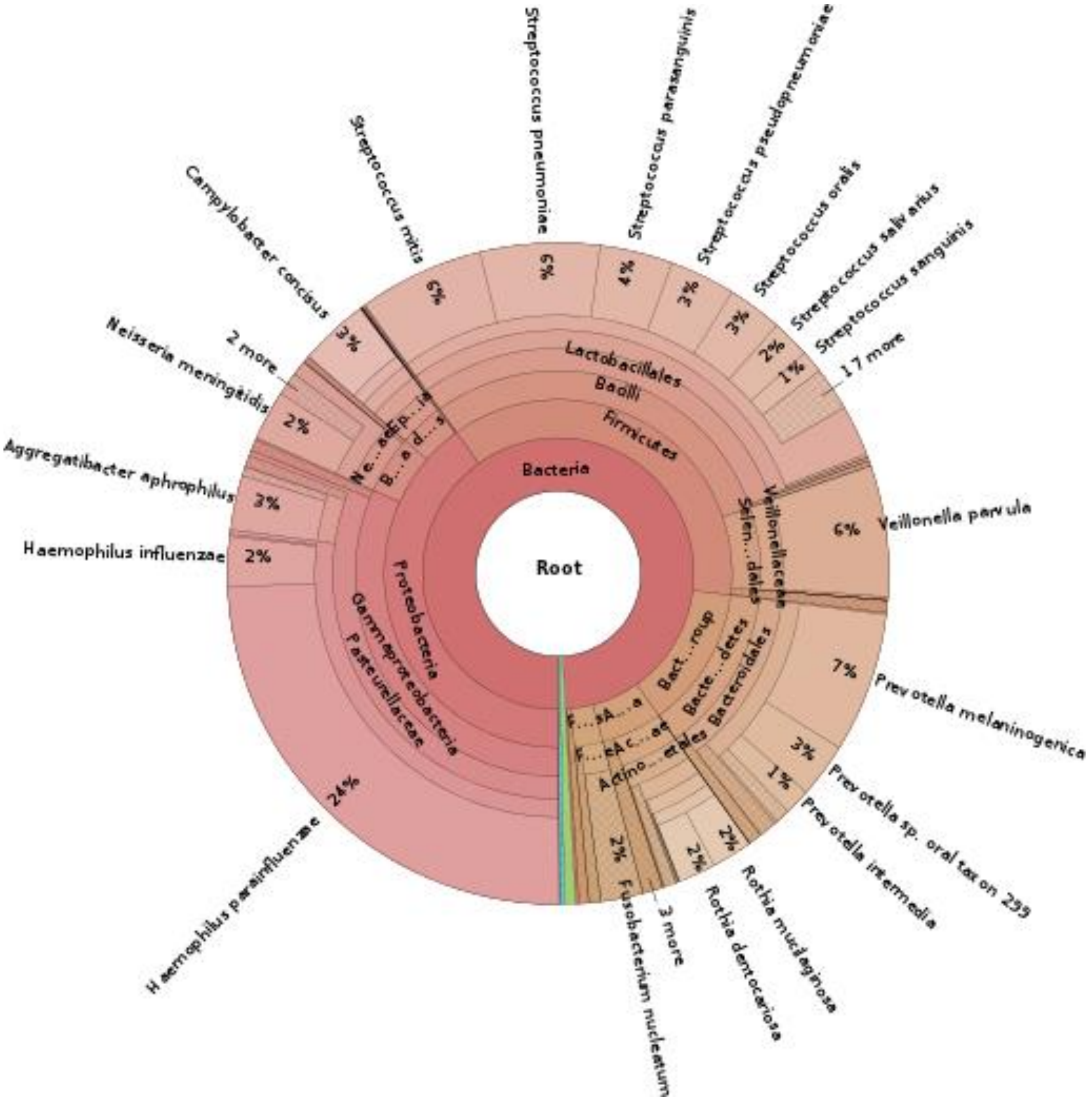

Figure S2: Taxonomic distribution of classified reads from SRA accession SRS014468.

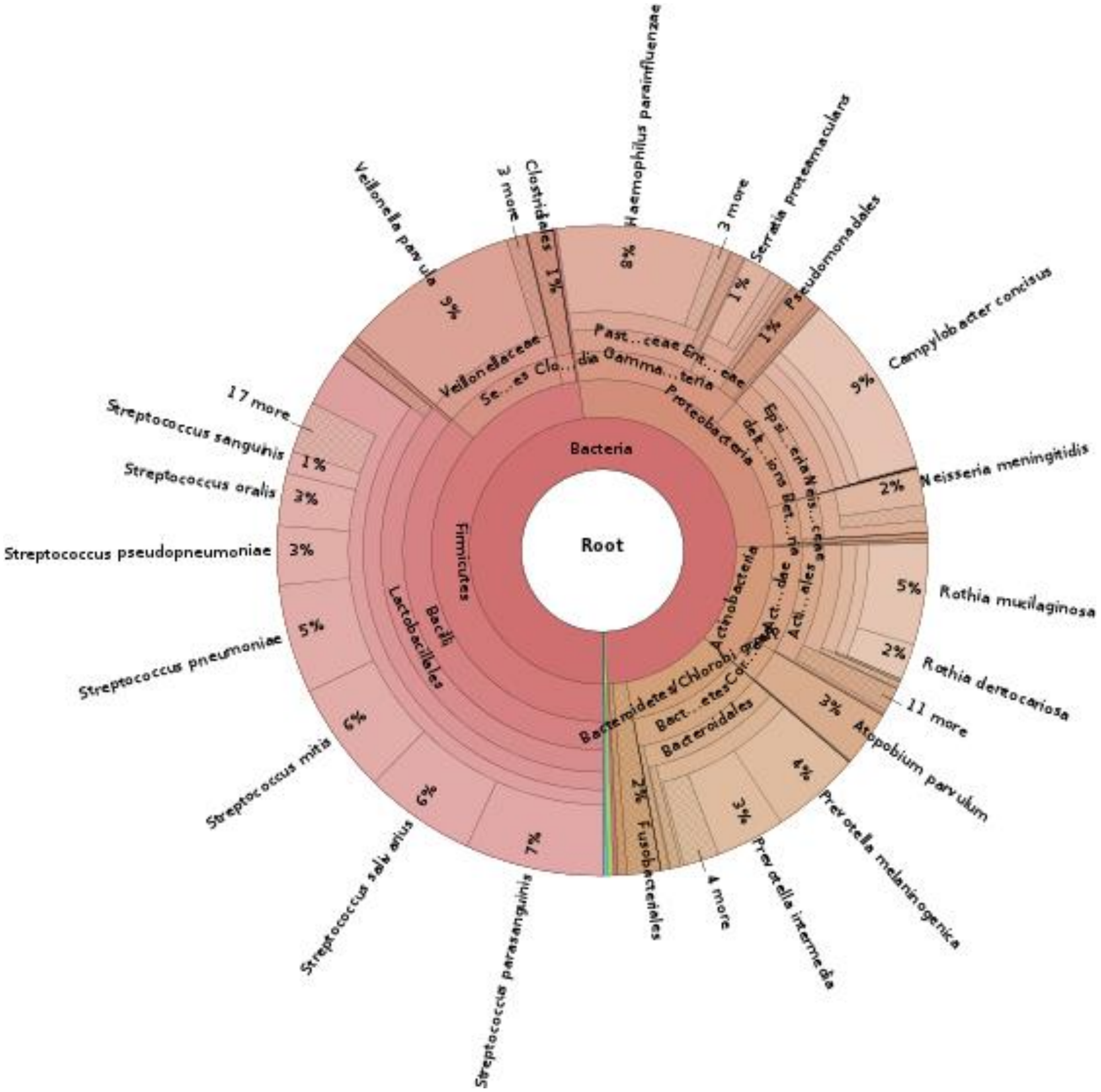

Figure S3: Taxonomic distribution of classified reads from SRA accession SRS015055.
